# Methylene blue is a potent and broad-spectrum inhibitor against Zika virus *in vitro* and *in vivo*

**DOI:** 10.1080/22221751.2020.1838954

**Published:** 2020-11-03

**Authors:** Zhong Li, Yuekun Lang, Srilatha Sakamuru, Subodh Samrat, Nicole Trudeau, Lili Kuo, Natasha Rugenstein, Anil Tharappel, Lianna D'Brant, Cheri A. Koetzner, Saiyang Hu, Jing Zhang, Ruili Huang, Laura D. Kramer, David Butler, Menghang Xia, Hongmin Li

**Affiliations:** a Wadsworth Center, New York State Department of Health, Albany, NY, USA; b Division of Preclinical Innovation, National Institutes of Health Chemical Genomics Center, National Center for Advancing Translational Sciences, Rockville, Maryland, USA; c The Neural Stem Cell Institute, Rensselaer, NY, USA; d Department of Biomedical Sciences, School of Public Health, University at Albany, Albany, NY, USA

**Keywords:** Methylene blue, flavivirus, Zika virus, Dengue virus, antiviral, protease inhibitor

## Abstract

Many flaviviruses including the Dengue virus (DENV), Zika virus (ZIKV), West Nile virus, Yellow Fever virus, and Japanese encephalitis virus are significant human pathogens, unfortunately without any specific therapy. Here, we demonstrate that methylene blue, an FDA-approved drug, is a broad-spectrum and potent antiviral against Zika virus and Dengue virus both *in vitro* and *in vivo*. We found that methylene blue can considerably inhibit the interactions between viral protease NS3 and its NS2B co-factor, inhibit viral protease activity, inhibit viral growth, protect 3D mini-brain organoids from ZIKV infection, and reduce viremia in a mouse model. Mechanistic studies confirmed that methylene blue works in both entry and post entry steps, reduces virus production in replicon cells and inhibited production of processed NS3 protein. Overall, we have shown that methylene blue is a potent antiviral for management of flavivirus infections, particularly for Zika virus. As an FDA-approved drug, methylene blue is well-tolerated for human use. Therefore, methylene blue represents a promising and easily developed therapy for management of infections by ZIKV and other flaviviruses.

## Introduction

The genus *Flavivirus* is composed of more than 70 viruses. Many flaviviruses cause serious and deadly human diseases. The four serotypes of Dengue virus (DENV), yellow fever virus (YFV), West Nile virus (WNV), Zika virus (ZIKV), Japanese encephalitis virus (JEV), and tick-borne encephalitis virus (TBEV) are categorized as global emerging pathogens [1,2]. The World Health Organization (WHO) has estimated the annual number of human cases at more than 390 million, 200,000, and 68,000 for DENV, YFV, and JEV, respectively. Approximately 3.9 billion people are at risk of DENV infection, which can lead to severe Dengue hemorrhagic fever and death [3]. Significant outbreaks of ZIKV, an emerging mosquito-borne flavivirus, initially occurred at Yap Island in 2007, French Polynesia in 2013, Easter Island in 2014, and most recently in Brazil in 2015 [4,5]. The virus quickly emerged in many new territories such as the UK, Canada, USA, etc. [2,6–8], presumably due to global travels. In addition to transmission via mosquito bite, ZIKV can be transmitted through sexual activities and blood transfusions [9–12]. Importantly, increasing evidence suggests that ZIKV infections are linked to Guillain-Barré syndrome, as well as an increase in babies born with microcephaly [4,13–15]. These associations strongly suggest that ZIKV infection during pregnancy can cause severe neurological damage in neonates. Although effective vaccines exist for YFV, JEV, and TBEV [16], there are currently no safe and effective vaccines for WNV and ZIKV. Moreover, a DENV vaccine was recently approved by FDA for children between 9 and 16-year-old. However, it does not protect naïve children and may increase the risk of developing more severe Dengue disease due to antibody-dependent enhancement [17,18]. Furthermore, due to the dangers and difficulties inherent in mass vaccination of large at-risk populations, it is desirable to be able to treat severe flavivirus infections with antiviral therapeutics that can be administered shortly after infection. However, such therapeutics are not yet available, despite the critical need for their development.

The flavivirus NS2B-NS3 protease is a highly conserved and replication-critical enzyme, composed of two viral proteins, NS3 protease and NS2B as a cofactor [19–23]. The function of this viral protease complex is to work together with host proteases to cleave the polyprotein precursor (PP) produced by the viral genome [19–23]. The flavivirus protease is a trypsin-like serine protease that preferentially cleaves protein substrates at sites immediately following two basic residues (K or R at positions P2 and P1) [3,21,24–26]. NS2B binding is required for NS3 function; mutations that abrogate NS2B binding greatly reduce the proteolytic activity of the complex [26–28].

Previously, we developed high-throughput screening assays to screen flaviviral protease inhibitors [24–26]. Using these assays, we identified a number of existing drugs as potent protease inhibitors blocking the interactions between viral NS3 protease and its cofactor NS2B[24,26]. Several of the hit drugs were characterized with not only *in vitro* protease inhibition activity but also *in vivo* antiviral efficacy [24].

**Figure 1.**
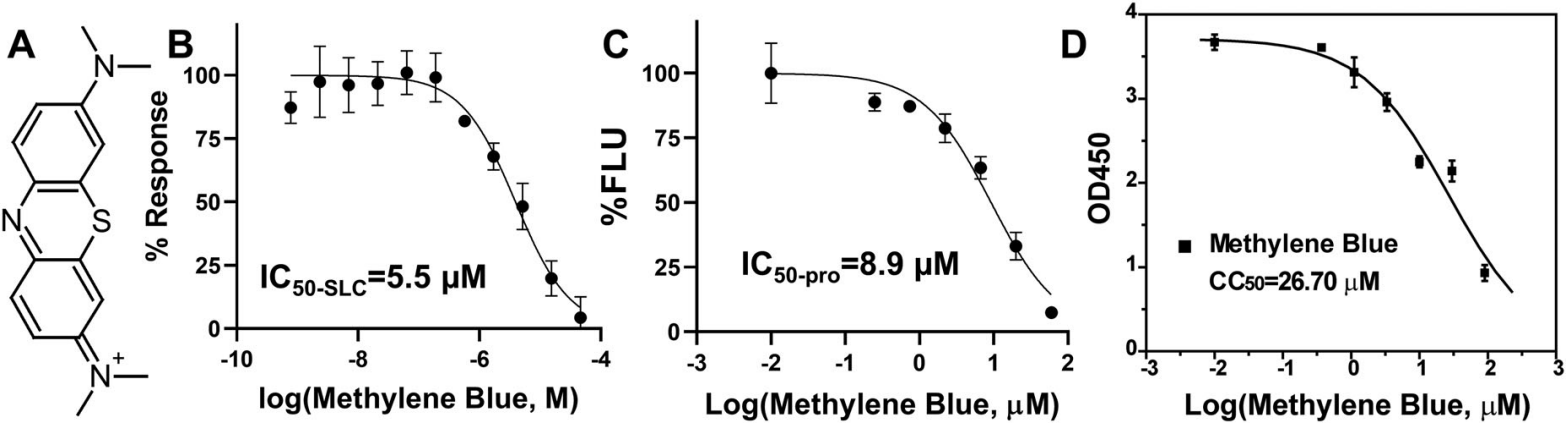
Inhibition of the NS2B-NS3 interactions and protease activity. (A) Chemical structure of MB. (B) Dose-dependent inhibition of SLC upon binding of NLuc-NS2B_49-66_ to GST-CLuc-NS3 of DENV2 by MB. *N* = 3. (C) Dose-response inhibitions of the DENV2 His-NS2B/His-MBP-NS3 protease activity by MB. *N* = 3. (D) Cell viability assay. A549 cells were incubated with various concentrations of MB and then assayed for viability at 48-hr post-incubation. *N* = 3.

In this study, we evaluated an FDA-approved drug, methylene blue (MB) (Figure 1(A)), as a potent Zika virus inhibitor. MB was found to significantly inhibit the interactions between NS2B and NS3 and also moderately inhibited the NS2B-NS3 protease activity, with IC_50_ values in micromolar range. MB significantly reduced titres of multiple strains of ZIKV and DENV2 with low micromolar and nanomolar EC_50_s in cell-based antiviral assays. In addition, MB was found to inhibit viral replication in cells relevant to ZIKV pathogenesis, and protect 3D mini-brain organoids from ZIKV infection. Moreover, MB treatment significantly reduced viremia *in vivo* in a mouse model. Furthermore, MB was found to inhibit viral replication in both entry and post-entry steps. Overall, the data suggested that MB is an effective and broad-spectrum inhibitor against ZIKV and DENV.

## Results

### MB inhibits NS2B-NS3 interactions

In a previous study [24], we developed a highly sensitive assay based on split luciferase complementation (SLC) to detect molecular interactions between viral protease NS3 and its co-factor NS2B. The SLC assay is composed of a DENV2 GST-CLuc-NS3 fusion protein and a His-NLuc-E66stop fusion protein which contains amino acids 49–66 of the DENV2 NS2B co-factor. Using the SLC assay, we identified and characterized several potent inhibitors against the viral NS2B-NS3 protease [24,26]. In the current study, using this assay, we found that MB significantly inhibited the interaction between the viral NS3 protease and its cofactor NS2B with an IC_50-SLC_ of 5.5 µM (IC_50-SLC_/IC_50-pro_/EC_50_/CC_50_ was defined as compound concentration required to reduce 50% of the SLC signal, protease activity, virus growth, and cell viability, respectively) (Figure 1(B), Table 1).

**Table 1.** IC_50_,^a^ EC_50_, CC_50_ (all in µM)

IC_50-SLC_ IC_50-Pro_			CC_50_						EC_50_					
	DENV2		A549	A549-ZIKV	HPEC	HPEC-ZIKV	HNPC	HNPC-ZIKV	DENV2	PRV^b^	MR^b^	FSS^b^	Mex^b^	BeH^b^
MB	5.5	8.9	26.7	6.4	6.9	46	4.3	29	0.36	0.087	0.2	0.14	0.087	0.14

^a^ *IC_50-SLC_*/*IC_50-pro_*/*EC_50_*/*CC_50_*: Compound concentrations required to reduce 50% of the split luciferase complementation (SLC) signal, protease activity (pro), virus production, and cell viability, respectively. All numbers are in µM. ^b^PRV, PRVABC59; MR, MR766; FSS, FSS13025; Mex, Mex I-44; BeH, BeH 819015.

### MB inhibits the NS2B-NS3 protease activity

We next performed dose-dependent protease inhibition studies on MB. The Protease inhibition assay used a synthetic quenched fluorescent peptide TAMRA-RRRRSAG-QXL570. Protease digestion leads to release of the QXL570 quencher, resulting in fluorescence increase. Addition of protease inhibitors abolishes the fluorescence increase. Using the quenched TAMRA peptide and an *in vitro* expressed DENV2 MBP-NS3 fusion protein and its core co-factor NS2B of DENV2 as described previously [24,26], we found that MB inhibited the viral NS2B-NS3 protease function with an IC_50-pro_ of 8.9 µM (Figure 1(C), Table 1).

TAMRA fluorescence may be quenched by compound addition. To rule out this possibility, we synthesized a quencher-free version of the peptide, TAMRA-RRRRSAG. Using this quencher-free peptide, we first found that MB at 7.5 µM did not have any significant impact on fluorescence of TAMRA (2 µM) (Supplemental Figure 1A). We next carried out a dose–response experiment. Our results showed that MB could quench the TAMRA fluorescence, but only at very high concentrations with an IC_50_ of 41 µM (Supplemental Figure 1B). In contrast, our protease inhibition assay showed that MB inhibited the NS2B-NS3 protease function with a much lower IC_50_ value (8.9 µM). The results indicated that the observed inhibition of NS2B-NS3 protease activity by MB was not resulted from MB-fluorophore effect but mediated by its interactions with NS2B-NS3 protease.

### MB is a highly potent inhibitor against DENV

We next measured the compound cytotoxicity using the WST-8 method as we described previously [24]. We measured the viability of A549 cells in the presence of a range of MB concentrations (Figure 1(D)). MB demonstrated moderate cytotoxicity towards A549 cells, with a cytotoxicity *CC_50-MB-A549_* of 26.7 µM (Figure 1(D), Table 1).

**Figure 2.**
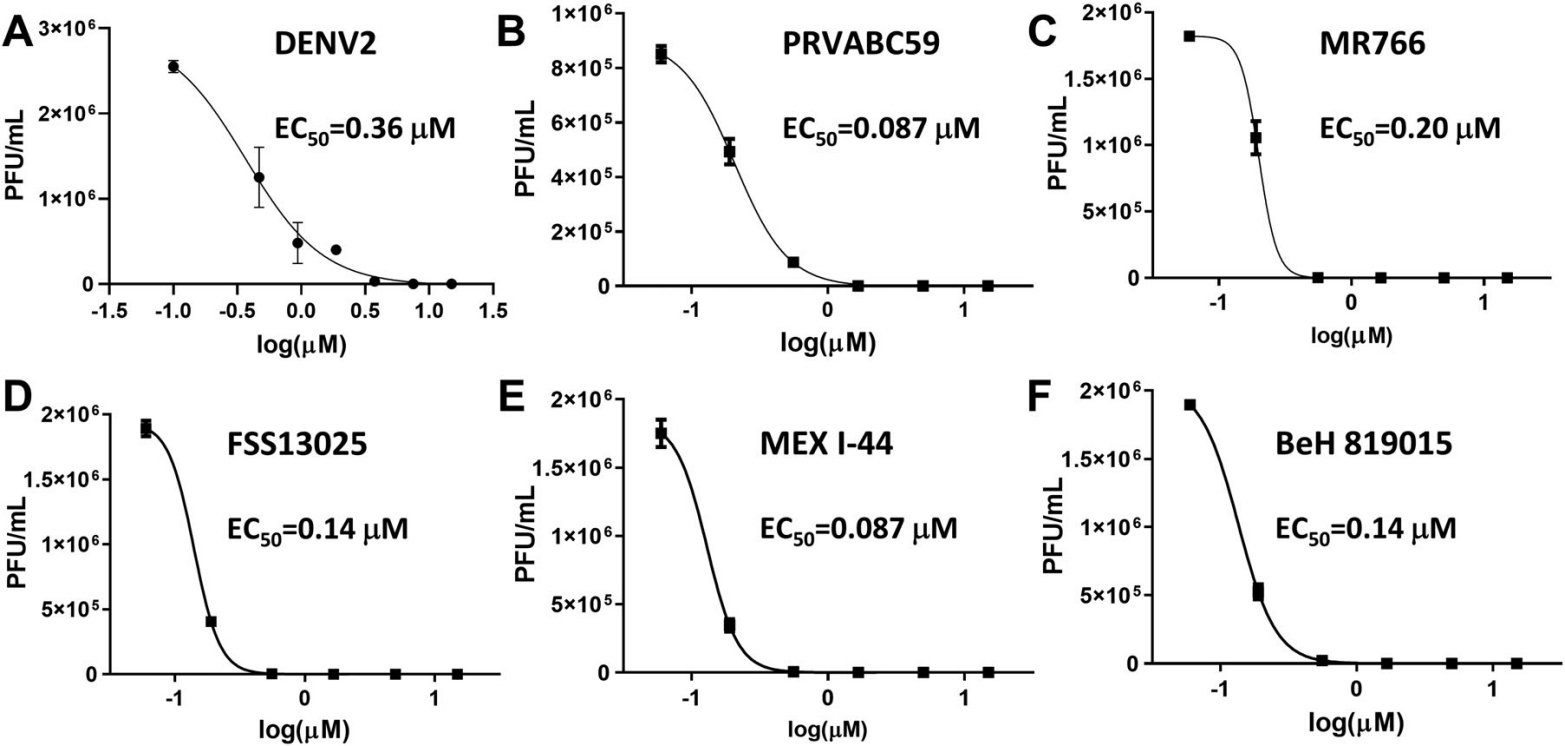
MB is a potent broad-spectrum inhibitor of representative flaviviruses. (A-F) Dose-dependent inhibition of representative flaviviruses DENV2 (A), and ZIKV strains PRVABC59 (Human/2015/Puerto Rico) (B), Mex I-44 (Mosquito/2016/Mexico) (C), MR766 (Rhesus/1947/Uganda) (D), BeH819015 (Human/2015/Brazil) (E), FSS13025 (Human/2010/Cambodia) (F) by MB in A549 cells. Viral plaque reduction assay was used. *N* = 3.

Having determined a concentration range at which the A549 cells were viable, we subsequently performed viral reduction assays to determine if MB was an effective inhibitor via reducing viral titres in cell culture. The EC_50_ value of MB was determined to be 0.36 µM for DENV serotype 2 (DENV2) (Figure 2(A), Table 1). Overall, MB demonstrated excellent therapeutic windows as reflected by a high therapeutic index of 51 (defined as CC_50_/EC_50_).

### MB inhibits multiple strains of ZIKV

 MB was previous found to inhibit WNV; however, this inhibition was through a photoactivation mechanism [29]. To investigate if MB inhibits additional flaviviruses and if inhibition requires photoactivation, we evaluated whether MB inhibited ZIKV without any light exposure. As shown in Figure 2(B–F), MB significantly inhibited all ZIKV strains tested (including the African strain MR766 and contemporary strains such as Brazil BeH, Puerto Rico PRVABC59, Cambodia FSS13025, and Mexico MeX I-44), with EC_50s_ in the low nanomolar range (Table 1).

**Figure 3.**
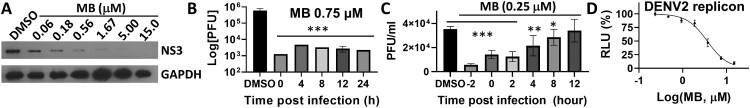
Mechanism of action studies. (A) WB analysis of dose response inhibition of ZIKV NS3 expression by MB. ZIKV-infected cell lysates were analysed using Western blot with anti-ZIKV NS3 (GTX133309, GeneTex, Inc.) and anti-GAPDH (CB1001, EMD Millipore) as primary antibodies. (B) 48-hour time of addition. MB (0.75 µM) was added at indicated time points post-infection. Viral titres were quantified using plaque forming assay 48 h post-infection. DMSO was added to control. *N* = 3. ***, *p*<0.001. (C) 24-hour single life cycle time of addition. MB (0.25 µM) was added at indicated time points. Viral titres were quantified using plaque forming assay 24 h post-infection. DMSO was added to control. *N* = 3. ***, *p*<0.001; **, *p*<0.005; *, *p*<0.05. (D) Dose-dependent inhibition of DENV2 replicon by MB. *N* = 3.

In addition to the viral plaque reduction assay, we also chose ZIKV to evaluate the inhibitions of viral RNA synthesis and viral protein expression by MB. As shown (Figure 3(A), left panel), the ZIKV RNA copy number was also significantly reduced in A549 cells in a dose-dependent manner by MB. Moreover, using a pan flavivirus anti-E antibody 4G2, an immunofluorescence assay (IFA) assay indicated that MB treatment also greatly reduced ZIKV antigen expression in A549 cells in a dose-dependent manner (Figure 3(B), top panel). It is noted that at higher MB concentrations, cell nuclei appeared lower than those at lower concentrations of MB and the DMSO control. It is possible that MB treatment together with viral infection causes more cytotoxic effect on A549 cells than MB alone. Using ImageJ [30], we quantified the numbers of nuclei under each conditions and calculated *CC_50-MB -A549-ZIKV_* as 6.4 µM (Figure 3(C), Table 1).

Nevertheless, these experiments confirmed that MB inhibits viral infectivity, viral RNA replication, and viral protein production, and is a broad-spectrum antiviral for flaviviruses.

### MB treatment leads to reduced NS3 protein production in cell-culture

We performed Western blot (WB) analysis of processed NS3 protein levels following MB treatment, using an NS3-specific antibody as we described previously [24–26]. Our results showed that MB treatment resulted in reduced production of processed NS3 in a dose-dependent manner, compared to the DMSO control (Figure 3(A)). The result could implicate that MB acts as a protease inhibitor to inhibit the polyprotein processing, leading to a reduced quantity of processed NS3 protein. Alternatively, the reduced production of NS3 protein could result from a reduction of viral replication by MB treatment, which leads to an overall reduction of viral particles and viral proteins.

### MB is effective at both entry and post infection steps

Antiviral inhibitors are generally divided into two categories, entry and post-entry (replication), respectively. Inhibitors targeting the viral protease complex should work in post-entry. To verify this, we first performed a 48-hour time-of-addition experiment by adding MB at different time points post-infection, followed by evaluation of viral plaque units at 48 h post-infection (Figure 3(B)). Our results showed that MB was equally effective in reducing ZIKV titre even at 24 h post-infection, leading to 2 log order of reduction of ZIKV. The results suggest that MB is equally effective in post-infection.

It was known that flavivirus complete one replication cycle in cells in about 24 h [31]. In order to further investigate the mechanism, we performed a 24-hour single viral life cycle time-of-addition experiment, using a lower concentration of MB (0.25 µM) (Figure 3(C)). Virus plaque production was quantified at 24-hour post-infection. Our results showed that MB (0.25 µM) was most effective at 2-hour pretreatment, leading to 85% reduction of viral plaque forming units. In addition, although less efficient than pretreatment, MB treatment at 2-hour post infection is equally effective as time 0 treatment, resulting in 60-65% viral plaque reduction (*p*<0.001). MB treatments at 4- and 8-hour post infection also significantly reduced viral plaque productions by 40% (*p*<0.005) and 20% (*p*<0.05), respectively. MB treatment at 12-hour post-infection was not effective in reduction of virus plaque production. The results indicate that MB has multiple modes of actions. Firstly, the notion that MB pretreatment is most effective in viral plaque reduction is in agreement with results from previous reports indicating that MB pretreatment of flavivirus greatly reduced virus infectivity through a photo inactivation mechanism [29–33]. Secondly, MB treatment is also effective up to 8-hour post-infection. The results suggest that MB also inhibits virus replication in post-entry step, which is consistent with our results that MB inhibits viral protease function.

### Inhibition of DENV2 replicon

To further investigate the nature of inhibition, we used a BHK-21 cell line stably expressing a DENV2 Renilla luciferase (Rluc) reporter replicon that does not require viral entry for viral replication [34]. Our results indicated that MB also effectively inhibited the DENV2 replicon replication with an EC_50-replicon_ of 3.6 µM (Figure 3(D)). The results confirmed that MB reduced viral replication at a post-entry step, which is again consistent with its mechanism of action as a protease inhibitor.

### MB inhibits viral production in cells relevant to ZIKV pathogenesis

**Figure 4.**
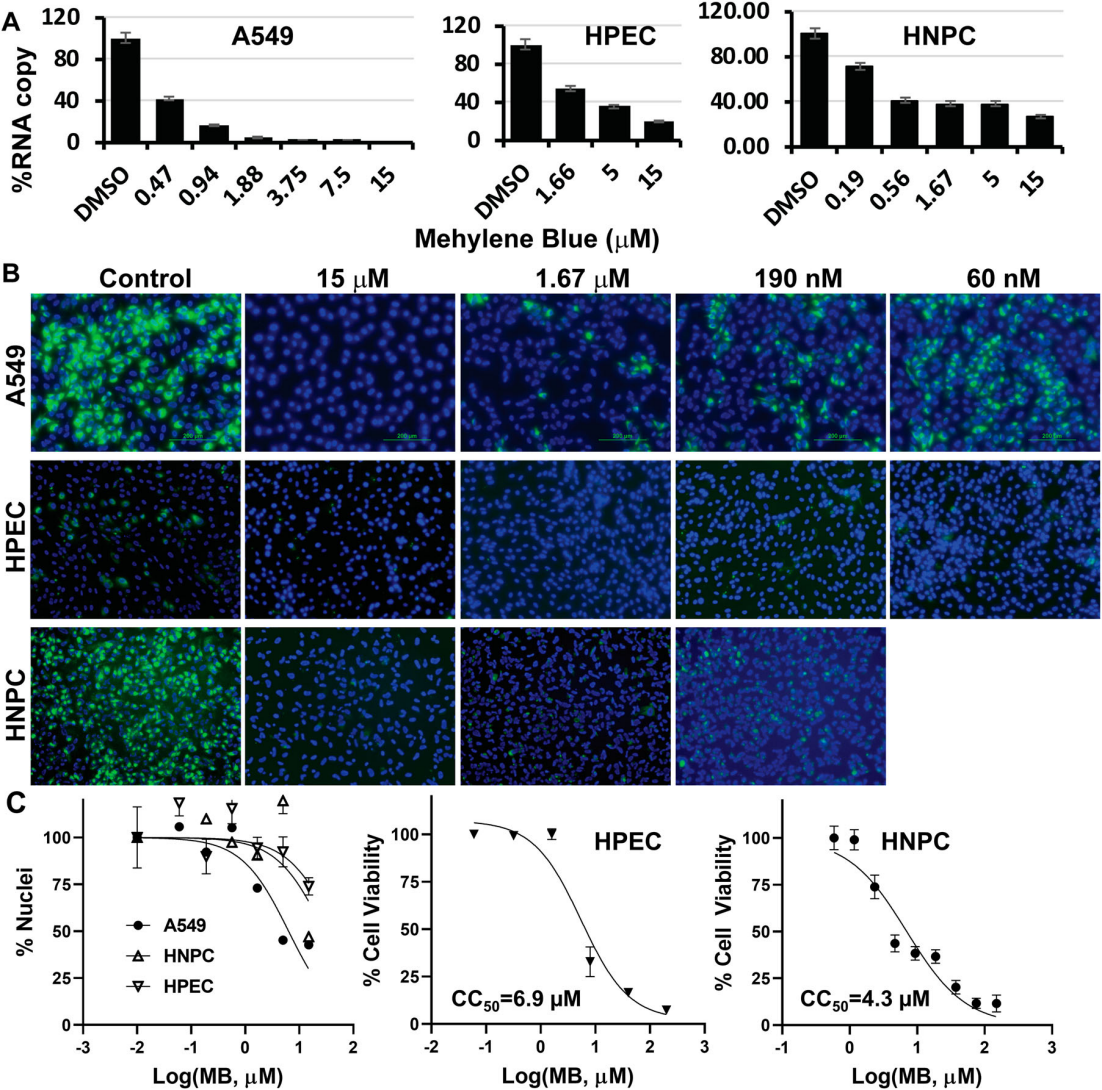
Inhibition of ZIKV in cells relevant to ZIKV. (A) qRT-PCR analyses of inhibition of viral RNA from ZIKV infected cells (A549, human placental epithelial cell (HPEC), and human neural progenitor cell (HNPC)) by MB. *N* = 3 (B) Immunofluorescence assay (IFA) of inhibition of viral protein expression by MB, using pan-flavivirus anti-E 4G2 antibody (green) (ATCC). Nuclei (blue) was stained in all IFA assays by the Hoechst stain solution. (C) Cell counting and viability assay. Left, A549, HPEC, or HNPC cells were incubated with various concentrations of MB, and then cell nuclei were counted by ImageJ. Middle and right, HPEC, or HNPC cells were incubated with various concentrations of MB in the absence of viral infection and assayed for viability at 48-hr post-incubation. *N* = 3. CC50s were determined by nonlinear regression of cell nuclei numbers or the WST-8 staining readings under each concentration.

To investigate whether MB is effective in human primary cells demonstrated to be relevant to ZIKV pathogenesis [35–39], we investigated inhibition of ZIKV by MB in human placental epithelial cells (HPECs) that derived from the inner surface of the amnion, and in human induced pluripotent stem cell (iPSC)-derived neural progenitor cells (HNPC) [40]. Our results showed that MB effectively inhibited ZIKV in HPEC and HNPC in dose-dependent manners (Figure 4(A), middle and right panels). Moreover, results from IFA indicated that viral protein expression was also drastically decreased in these ZIKV-relevant cells (Figure 4(B), middle and bottom panels).

We further measured the cell viability of HPEC and HNPC upon MB treatment in the presence and absence of ZIKV infection (Figure 4(C)). In the absence of ZIKV infection, MB has moderate cytotoxicity to HPEC and HNPC, with *CC_50_* of 6.9 and 4.3 µM, respectively (Figure 4(C), middle and right panels). Interestingly, *CC_50_* calculated for MB from numbers of nuclei for HPEC and HNPC in the presence of ZIKV are much higher than those for MB alone (Figure 4(C), left panel; Table 1). Nonetheless, the estimated *EC_50_* values of MB for ZIKV infection of these cells are much less than the *CC_50_* values, regardless of viral infection.

Overall, these experiments demonstrate that MB is an effective antiviral in placental and neural progenitor cells relevant to ZIKV infection.

### Generation of an infectious clone of ZIKV expressing Venus fluorescence protein (ZIKV-zVenus)

In order to directly monitor the effect of MB on virus propagation, we generated a full-length infectious ZIKV cDNA clone expressing a fluorescent reporter gene Venus, using a strategy similar to that described by Schwarz et al. [41]. The DNA sequence encoding the mVenus reporter gene was modified based on the calculated codon usage of ZIKV and hence named zVenus. The ZIKV-zVenus can stably replicate in Vero cells suitable for antiviral studies.

### MB protects 3D mini-brain organoid from ZIKV infection

The risk of developing microcephaly, a birth defect where a baby has a smaller than normal head circumference, is significantly increased for newborns from mothers with ZIKV infection during pregnancy. Brain-related or other neurological complications are common problems for babies born with microcephaly. Recently, 3D cerebral organoids derived from neural stem cells were used to dissect ZIKV pathogenesis and anti-viral development [38,42–44]. Due to their unique features, the 3D brain organoids can better represent the composition, diversity and organization of cell types found in the developing human brain than the cultured 2D monolayer cells. Therefore, we used the 3D mini-brain organoid model to further investigate whether MB can protect against ZIKV-associated neurological damage.

**Figure 5.**
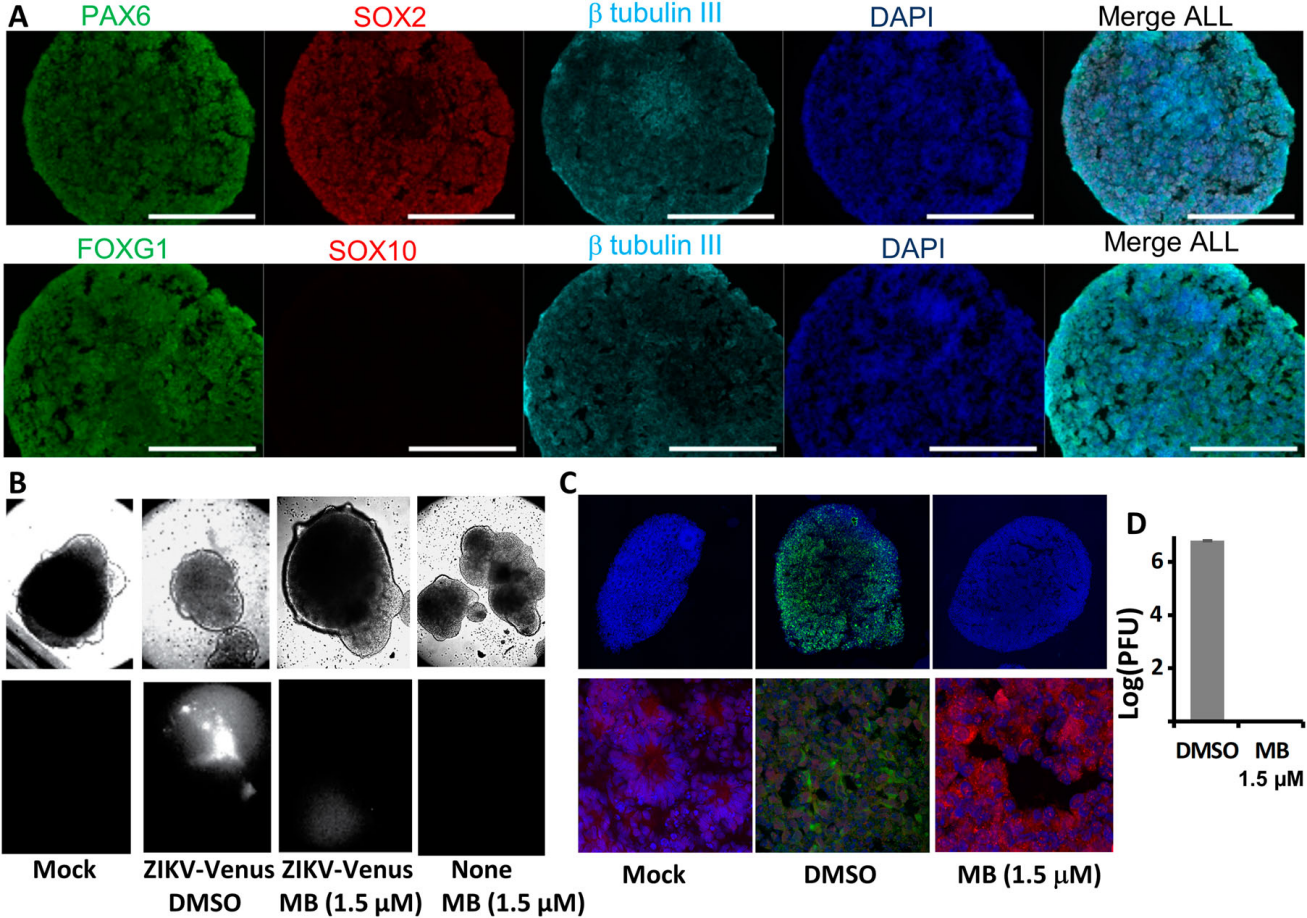
MB protects 3D mini-brain organoid from ZIKV infection. (A) Forebrain regional specification of organoids. Organoids were stained positive for forebrain identity markers PAX6 (green, upper panel), FOXG1 (green, lower panel) and SOX2 (Red (Magenta after merge with DAPI (Blue)), upper panel) at 20 days. The sections were stained positive for general neuronal marker TUJI (cyan, upper and lower panels) and were negative for SOX10 (Red, lower panel). Nuclei (DAPI, Blue); scale bar 200 μm. (B) ZIKV organoid infected with ZIKV-Venus. The 3D organoids were infected with PBS (Mock), or ZIKV untreated (DMSO), or ZIKV treated with MB (1.5 µM), or Mock treated with MB (1.5 µM). Upper panel, bright field image of intact organoids. Lower panel, Venus fluorescence image (excitation 515 nm, emission 528 nm) of the intact 3D organoids. (C) Slices of organoid infected with ZIKV PRVABC59. Organoids infected with ZIKV PRVABC59 treated with DMSO or MB. The 3D organoids were infected with either PBS (Mock), ZIKV untreated (DMSO), or ZIKV treated with MB (1.5 µM). Upper panel, IFA using anti-E 4G2 antibody (green); blue, DAPI. Lower panel, details of signature rosette region of the 3D organoids (Mock) or infected region (DMSO/ZIKV and MB). Red: Pax6. 4G2 antibody (ZIKV), green; DAPI, blue. (D) ZIKV production from the 3D organoids at 5 dpi. Culture supernatants were collected, and virus production was quantified by PFU assay. *N* = 3.

Using an established protocol, we used induced pluripotent stem cells (iPSC) derived from a healthy control (Male, Caucasian) to differentiate and generate region-specific organoids that were patterned to resemble the dorsal forebrain (Figure 5(A)) [45]. Using a panel of antibodies against forebrain identity markers PAX6 (dorsal forebrain progenitors; upper panel), FOXG1 (lower panel) and SOX2 (neural ectoderm marker, upper panel), we stained the organoids and demonstrated the 3D forebrain organoids were obtained. We also used a panel of antibodies to demonstrate that the organoids were positive for general neuronal marker TUJI (neuron-specific class III β-tubulin; upper and lower panels) and were negative for SOX10 (Neural crest, Red, lower panel).

At day 20, the organoids displayed signature features of forebrain, including neural rosettes (Figure 5(A–C), lower panel). We pre-treated the organoids with MB (1.5 µM) or DMSO control. At day 21, the organoids were infected with either Mock, ZIKV strain PRVABC59, or ZIKV-Venus, at an estimated MOI of 1 in the presence of 1.5 µM MB or DMSO control. ZIKV infection was evaluated by plaque forming unit (PFU) assay at 5 dpi and by fluorescence imaging at 7 dpi, respectively. Our results showed that MB at 1.5 µM did not have any toxic effect on the 3D organoids, which remained intact and unchanged in morphology (Figure 5(B), upper panel). Using Zika-zVenus, we showed that untreated organoids (DMSO) displayed significant fluorescence, whereas only very week fluorescence signal could be detected for organoids treated with MB (Figure 5(B), lower panel). Our results suggest that MB treatment significantly abolishes ZIKV infection in the 3D organoid.

To further investigate the antiviral protection of MB, the 3D mini-brain organoids were sliced for immunostaining (Figure 5(C)). As shown, untreated organoids (DMSO) were infected with ZIKV throughout all regions (Figure 5(C)). In contrast, MB treatment completely protected the organoids from ZIKV infection (Figure 5(C)). No virus could be found in the 3D organoid. Moreover, MB treatment completely inhibited ZIKV production, resulting in 6 log order of reduction of virus (Figure 5(D)). Collectively, the results indicated that MB is an effective inhibitor to protect developing human cortical tissue from ZIKV infection.

### MB reduces viremia in a ZIKV mouse model

**Figure 6.**
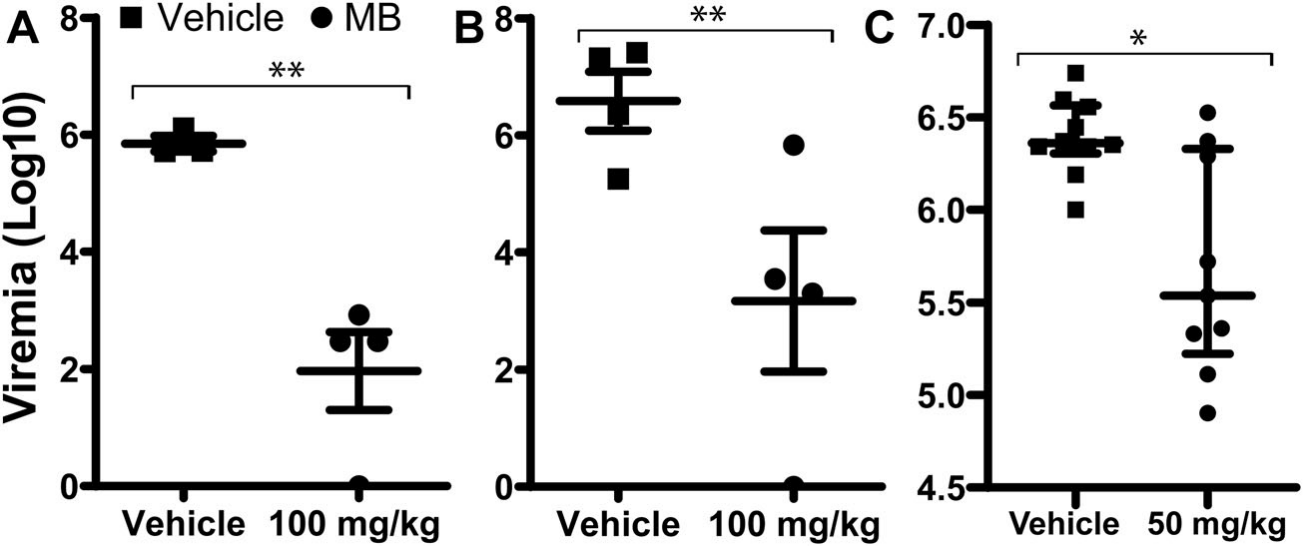
*In vivo* antiviral activity of MB against ZIKV. (A-C) Viremia was detected by plaque forming unit assay on day 3 post-infection of ZIKV with doses of 1.7 × 10^4^ (A) or 1.7 × 10^5^ (B-C) PFU/mouse in four-week-old A129 mice, which were treated with vehicle or MB though oral gavage. Difference between MB or vehicle treatment was analysed by using the unpaired, two-tailed T-test, *, *P*<0.05; **, *P*<0.005. *N* = 4 for A and B. For C, vehicle, *N* = 10; MB, *N* = 9.

Using an *IfnαβR*^-/-^ mouse model, we measured viremia reduction by MB treatment using a similar protocol as we described previously using the Balb/C viremia model [24]. We showed that MB treatment at 100 mg/kg/day significantly resulted in about 4 log orders of reduction in ZIKV-induced viremia in the *Ifn*αβ-/- mice inoculated with 1.7 × 10^4^ PFU PRVABC59 ZIKV/mouse compared to the vehicle control (Figure 6(A)). The experiment was repeated with a higher infection dose (1.7 × 10^5^ PFU PRVABC59 ZIKV/mouse), and similar results were obtained (Figure 6(B,C)).

For all these experiments, mice in both treated and untreated groups did not show any clinical sign of illness and body weight loss during the 4-day period (Supplemental Figure 2). The result is consistent with findings from our and others’ previous studies, indicating that the *IfnαβR*^-/-^ mice start to show clinical signs and body weight loss from day 5 or 6 post infection of ZIKV [24,46].

Overall, the results indicated that MB not only inhibited viral replication *in vitro* but also significantly reduced viremia in an *in vivo* animal model.

## Discussion

The majority of flaviviruses are significant human pathogens. Recent outbreaks of ZIKV and its implication in significant human disease highlight the need for the development of effective antivirals. Drug repurposing is the fastest way to develop new therapies. Therefore, in this study, we explored MB as a candidate orthosteric inhibitor blocking interactions between flavivirus NS2B and NS3. We showed for the first time that MB not only has novel anti-flaviviral activities, but also has a novel mechanism of action.

In this study, we found that MB is an inhibitor for flavivirus NS2B-NS3 hetero-protease complexes. MB inhibited the viral protease activities of DENV2 with micromolar efficacy *in vitro*. In cell culture, MB significantly inhibited the growth of representative flaviviruses, including DENV2 and various clinical ZIKV isolates, with EC_50_ in the nanomolar range. IFA and qRT-PCR analyses indicated that not only was virus growth inhibited by MB treatment, but viral RNA synthesis and protein production were also drastically reduced by MB treatment. Cytotoxicity analysis indicated that MB has large therapeutic window for these flaviviruses. In mechanistic studies, we showed that MB works at both entry and post-entry steps. We demonstrated that MB was most effective against ZIKV in 2-hour pre-infection, consistent with previous finding that MB can photo-inactivate West Nile virus, a virus within flavivirus family [33]. Time of additional studies also demonstrated that MB was very effective in reducing virus production up to 8-hour post infection in a single viral life cycle experiment or up to 24-hour post-infection in a multiple life cycle experiment, indicating that MB works in post-entry as well. In addition, our results showed that MB reduced NS3 protein production and inhibited DENV2 replicon replication, providing strong evidence that MB works in post-entry step. Moreover, we showed dose-dependent rescue by MB from viral infection in all ZIKV-relevant cell types. Furthermore, we found that MB could completely protect 3D cortical mini-brain organoids from ZIKV infection. The finding implies that MB can eliminate infection of ZIKV from human neural progenitor cells, and possibly minimize the risk of microcephaly resulting from ZIKV infection of pregnant women [47]. Finally, we showed that MB treatment significantly reduced viral viremia in the A129 ZIKV mouse model infected with clinical strain ZIKV PRVABC59. Overall, these results demonstrate that MB is a broad-spectrum inhibitor against flaviviruses both *in vitro* and *in vivo*.

As a water-soluble dye, MB has been used for a long time in industry and medicine [48]. As a drug, MB has excellent oral bioavailability (∼72%) [49]. MB is rapidly absorbed from the gastrointestinal tract and widely distributed throughout the body. Following oral administration of 500 mg MB, maximal plasma concentration is achieved at 2 h with a C_max_ of 12.2 µM and a plasma half-life of about 20 h [49]. MB is mainly eliminated through the renal excretion pathway [50,51]. As a registered drug with various brand names, although MB is not recommended for use in pregnant women, MB can be clinically used to treat non-pregnant patients in most countries for many medical applications, such as the prevention of ifosfamide-induced encephalopathy and urinary tract infections, treatment of methaemoglobinaemia, priapism, septic shock, vasoplegic syndrome, and malaria, and even as an experimental drug against Alzheimer’s disease [48]. In addition to these medical conditions, MB was also found to act as an antiviral for HIV and HCV infections [52,53]. In the late 19th and early 20th centuries, MB was most widely used as an antimalarial drug. It was well tolerated by human patients [54,55]. An oral dose of 1,000 mg per day has been previously applied to malaria patients for up to 30 days without significant side effects [48]. This clinically used dose is approximately equal to 14–17 mg/kg/day, assuming an average body weight of 60–70 kg for a human patient. In our experiment, we used a maximum oral dose of 100 mg/kg in the ZIKV mouse model. This mouse dose is equivalent to 8 mg/kg/day, assuming a mouse to human conversion factor of 12.3 [56]. The results suggest that MB is an effective option for human use to fight ZIKV and DENV infections.

In summary, MB, as an FDA-approved drug, has excellent bioavailability and safety profiles. Our findings establish the efficiency of MB to eliminate infections of ZIKV and DENV and provide a path forward to minimize the risk of foetal disease, Guillain-Barré syndrome, and potentially fatal severe Dengue disease.

## Experimental procedures

### Compounds

MB was purchased from Sigma-Aldrich (St. Louis, MO).

### Cloning, expression and Purification

All clones were generated as described previously [24]. All proteins of DENV2 were expressed in *Escherichia coli* strain Rosetta 2(DE3) (EMD Biosciences) and purified through a nickel-nitrilotriacetic acid (NTA) column (Qiagen) or Glutathione sepharose 4B (GE HealthCare), followed by a gel filtration 16/60 Superdex 200 column (GE HealthCare).

Split Luciferase Complementation (SLC) Assay.

The SLC assay was performed as previously described [24,26,57]. Briefly, MB was prepared in 11-point titrations with concentration ranging from 390 pM to 23 μM (3-fold dilution) in the SLC assay. The GST-CLuc-NS3 protein (80 nM final concentration) was dispensed into test buffer with MB, and incubated for 30 min. Then the NLuc-E66stop NS2B was added to the mixture to a final concentration of 80 nM. The D-luciferin substrate (Gold Biotechnology, Inc.) was added to a final concentration of 5 µg/ml. The reaction mixture was incubated to 2 h at room temperature and read using a ViewLux Plate Reader. IC_50-SLC_ values were determined by fitting dose-dependent titration points to the Hill equation.

### Protease substrate assays

The DENV2 NS3-MBP fusion protein (12.5 nM) was mixed with methylene blue (at various concentrations, or DMSO control) in reaction buffer (20 mM Tris pH 8.0, 100 mM NaCl, 5% Glycerol, and 0.05% CHAPS) and incubated at 4^°^ C for 30 min. Then the DENV2 His-NS2B was added at 1 µM final concentration. The peptide substrate TAMRA-RRRRSAG-GXL570 (Biomatik) was added to the mixture at a final concentration of 2 µM, and substrate cleavage was monitored over time at 37° C in a BioTek Flx800 at excitation/emission wavelengths of 520 nm/575 nm (TAMRA substrate). The rate of increase in RFU over time was calculated in the linear range and normalized as a percent of the DMSO control.

For MB quenching assay, quencher-free TAMRA-RRRRSAG peptide (Biomatik) was synthesized. The quencher-free TAMRA peptide (2 µM) was incubated with MB at a fixed (7.5 µM) or a concentration series (2-fold dilutions from 60 µM to 3.75 µM) in the same reaction buffer in triplicate at 22 °C for 1 h. Fluorescence intensities were recorded as described above. RFU was normalized as a percent of the DMSO control. IC_50-SLC_ value was determined by fitting dose-dependent titration points to the Hill equation, using GRAPHPAD 8.0 (San Diego, CA).

### Cytotoxicity assay

Cytotoxicity was measured by a WST-8 cell proliferation assay using a WST-8 cell proliferation assay (Dojindo Molecular Technologies, Inc.) as described previously [24]. The cytotoxic concentration CC_50_ was calculated using a sigmoidal nonlinear regression function to fit the dose–response curve using the GRAPHPAD 8.0 (San Diego, CA). For CC_50_ determination of viral infected cells in IFA, cell nuclei were counted using ImageJ and a nonlinear regression function was used to fit the dose–response curve using the GRAPHPAD 8.0 (San Diego, CA).

### Antiviral assay

A viral titre reduction assay was used to determine the compounds’ effect on selected flaviviruses, including DENV2 and various ZIKV strains, as we described previously [24–26]. Briefly, viral infection was done by inoculation of approximately 2 × 10^5^ cells with various viruses at MOI of 0.1 PFU/cell for A549 cells, or MOI of 1 PFU/cell for HPEC and HNPC cells in 24 well plates. Virus yields were quantified at 48-hour post-infection using standard viral plaque forming assay with Vero cells, as we described previously [24].

The effective concentration EC_50_ was determined by nonlinear regression fitting of the dose–response curve using the ORIGIN Suite 6.0 (Origin Lab, Wellesley Hills, MA) or GRAPHPAD 8.0 (San Diego, CA).

Human primary placental epithelial cells (HPECs) derived from the inner surface of the amnion were purchased from Cell Applications, Inc., and cultured according to manufacturer’s manual. Human HNPC, derived from iPSC generated using the STEMCCA Cre-Excisable Constitutive Polycistronic (OKSM) lentivirus, was purchased from EMD Millipore and cultured according to manufacturer’s manual. The antiviral efficacy experiments with HNPCs were carried out with a ZIKV MOI of 1 as described previously [24–26].

All cells were tested as free of Mycoplasma contamination.

### Immunofluorescence assay

The immunofluorescence assay was carried out as described previously [24–26]. Briefly, ZIKV-infected cells treated with DMSO or drugs stained with a mouse monoclonal pan anti-E antibody 4G2 (ATCC), a DyLight® 488 goat anti-mouse IgG (ImmunoReagents, Inc.), and nuclear staining dye Hoechst at 48-hour post-infection. Fluorescence images were recorded under a fluorescence microscope equipped with an Olympus DP71 imaging system.

### Quantitative qRT-PCR

50 µl of cell supernatant samples were extracted on an Applied Biosystems MagMAX Express-96 Deep Well Magnetic Particle Processor. TaqMan gene expression qRT-PCR assays were performed with 5 ul of the extracted RNA using the TaqMan One-step RT–PCR Master Mix Reagents Kit (PE Biosystems) on Applied Biosystems 7500 Real-time PCR System. TaqMan primers for ZIKV were CCGCTGCCCAACACAAG and CCACTAAYGTTCTTTTGCAGACAT with ZIKV probe Cy5-AGCCTACCT/TAO/TGACAAGCAGTCAGACACTCAA-IAbRQSp. Samples were analysed by relative quantification using the 2−ΔΔCT (“delta-delta Ct”) compared with the endogenous control.

### Western blot

Western blot was performed using anti-ZIKV NS3 (GTX133309, GeneTex, Inc.) and anti-GAPDH (CB1001, EMD Millipore) antibodies, as described previously [24,25]. A549 cells was infected with 10^6^ PFU/well in the presence of DMSO or MB at different concentrations. At 48 h post infection, cells were washed twice with PBS buffer and manually scraped out the 6-well plates. Cells were spun down and supernatant was discarded. Cells were treated with 30 µl of complete protease inhibitor cocktail in PBS, followed by addition of 30 µl of SDS-PAGE loading buffer. The mixtures were boiled at 95°C for 10 min and spun at 15,000 rpm for 10 min. Sample supernatants were analysed using 12% SDS-PAGE. Anti-ZIKV NS3 (GTX133309, GeneTex, Inc.) and anti-GAPDH (CB1001, EMD Millipore) were used as primary antibody.

### DENV2 replicon assay

BHK-21 cells stably expressing DENV2 replicon with a *Renilla luciferase* (*Rluc*) reporter gene [34] were seeded into white 96-well plate at a density of 2 × 10^5^ cells per well. The cells were incubated at 37°C in a humidified incubator with 5% CO_2_ for 24 h in a 100 µl medium containing Minimal Essential Medium (MEM) supplemented with 10% foetal bovine serum (FBS), 100 I.U./ml penicillin, 100 μg/ml streptomycin, and 300 μg/ml G418. Upon 24 h incubation, culture medium was discarded. Fresh culture medium of 100 µl with a concentration series of compounds or DMSO control was added to the cells in triplicates. The culture was further incubated for 48 h. The cells were then washed twice with 100 µl PBS, followed by addition of 25 µl of a lysis buffer containing 1X PBS and 1% Triton X-100. The mixture was incubated at room temperature with gentle shaking (50 rpm) for 30 min. Assay buffer (125 µl) containing 1X PBS, 0.05% CHAPS, and 0.1% BSA was then added to each well. In the meantime, a fresh 4X working coelenterazine substrate of was prepared by diluting 1 µl of stock coelenterazine (2.4 mM) dissolved in ethanol into 10 ml assay buffer (10,000-fold dilution). Finally, 50 µl 4X coelenterazine substrate was added to each well with a final substrate concentration of 0.06 µM, using a Veritas luminometer. The luminescence was recorded immediately using the Veritas luminometer. The luminescence data was normalized to DMSO control. The EC_50_ was determined by nonlinear regression fitting of normalized experimental data in GraphPad Prism 8.0.

### Culturing of human iPSCs

Human iPSC F11350.1 culture was maintained in feed-free conditions using mTeSR1 medium (StemCell Technologies #85851) with 5X supplement (StemCell Technologies #85852). Cells were maintained on six-well plates coated with human embryonic stem cell-qualified Matrigel at a 1:60 dilution (Corning Catalog #354277) and passaged with ReLeSR (StemCell Technologies #05872). Cortical 3D organoids were generated based on the protocol described by Yoon et al. [45]. Briefly, iPSCs were dissociated with accutase (ThermoFisher #A1110501) and then plated at a density of 3 × 10^6^ cells per well in AggreWell 800 24-well plate (StemCell Technologies #34811) in Essential 8™ medium (ThermoFisher #A1517001) supplemented with 10 µM ROCK inhibitor (Tocris #Y27632). The plate was centrifuged at 100 x g for 3 min and incubated at 37°C overnight. The next day (culture day 0) the organoids were transferred to a low attachment 10 cm plate (Corning #3262) and maintained in 10 mL of neural induction medium: Essential 6™ medium (Life Technologies, #A1516401) supplemented with two SMAD pathway inhibitors (10 µM SB431541 (R&D #1614/50) and 2.5 µM dorsomorphin (Tocris #3093)) and Wnt pathway inhibitor 2.5 µM XAV-939 (Tocris #XAV-939). Media were exchanged daily. On day 6, the media was changed to neural expansion medium: Neuralbasal A medium (ThermoFisher #10888) supplemented with 2% B27 (without Vit A; ThermoFisher #12587001), 1% antibiotic-antimycotic (ThermoFisher #15240062), 1% glutaMAX (ThermoFisher # 35050061), 20 ng/mL FGF2 (R&D #233-FB-500), and 20 ng/mL EGF2 (PeproTech #AF-100-15). Media were exchanged daily.

### Cryopreservation

Organoids were fixed in 4% paraformaldehyde (Santa Cruz) overnight at 4°C. They were then washed three times in PBS and transferred to 30% sucrose solution for 72 h. Organoids were then transferred into plastic cryomold (10 X 10 X 5mm; Tissue Tek cat no 4565) with embedding medium (Tissue-Tek OCT compound, Sakura Finetek 4583) and stored at −80°C. For immunohistochemistry, 20 μm thick sections were cut with a Leica cryostat (model CM3050S).

### Immunohistochemistry

Cryosections were blocked in 3% bovine serum albumin (BSA), 10% normal goat serum (NGS), and 0.3% Triton X–100 diluted in PBS for 1 h at room temperature. The sections were incubated overnight at 4°C with primary antibodies diluted in blocking solution. Sections were washed three times with PBS and then incubated with appropriate secondary antibodies diluted in blocking solution for 1 h. The following primary antibodies were used for immunohistochemistry: anti-Pax-6 (1:200; Biolegend #901301), anti-Sox2 (1:100; Santa Cruz #sc-365823), anti-FoxG1 (1:500; Takara #M227), Sox10 (1: 100; Santa Cruz # sc369692), and anti-tubulin III (1:1000; Sigma #T8660).

### Generation of an infectious clone of ZIKV expressing Venus fluorescence protein (ZIKV-zVenus)

To generate a ZIKV carrying a reporter gene, we developed an infectious cDNA clone from a ZIKA HOND 2016 (GenBank Accession # KX906952 [58]) which was isolated by the New York State Department of Health Arbovirus laboratory. This infectious cDNA clone driven by CMV promoter was based on the design reported by Schwarz et al. [41]. An intron sequence was positioned in an NS1 gene to stabilize the full-length single plasmid construct. The coding sequence of fluorescence reporter gene mVenus was modified using the calculated codon usage of the viral genes and inserted between NS1 and NS2, where insertion of a foreign gene is tolerated[59]. This virus, named ZIKV-zVenus, grew to high titres in Vero cells. A genetic marker with 3 silent nucleotides is placed in the E gene; these changes resulted the creation of a Bam HI site while simultaneous abolishing an Avr II.

### *In vivo* protection efficacy

All animal studies involving infectious ZIKV were conducted at an Animal Biosafety Level 2 (ABSL-2) facility at the Wadsworth Center with Institutional Biosafety Committee approval. The *in vivo* antiviral activity of MB was evaluated in a viremia animal model.

A group of four-week-old A129 mice were infected by subcutaneous injection with 1.7 × 10^4^ or 1.7×10^5^ PFU of the PRVABC59 strain. Then, the infected mice were administered, through oral gavage, with MB at 100 mg/kg or 50 mg/kg of body weight (*N* = 4, or *N* = 9) or with vehicle control (*N* = 4, or *N* = 10) every day for 3 consecutive days post-infection (dpi). Mice were observed daily for signs of illness and mortality. A clinical score system was established as follows: 0, normal activity and weight. No overt signs of disease. 1, if any of these is observed: hunched posture; ungroomed, lumped, or ruffled fur; ataxia (no paralysis); decreased righting response; or 15% weight loss (Food and water must be accessible from cage floor); Piloerection. 2, paralysis of 1 limb (any limb), but animal otherwise in good physical condition. Food and water must be accessible from cage floor. 3, paralysis of any 2 or more limbs; paralysis of 1 limb (any limb), but this limb also shows wounding/self-biting; Laboured breathing and unresponsiveness (animal may self-isolate from cage mates); or ≥ 20% weight loss. 4, moribund; animal stretched out on cage floor and/or unresponsive. 5, dead. Mice scored combined score of 3 or above will be euthanized. Viremia on day 3 post-infection (pi) was determined by plaque forming assay, and statistical analysis was performed using the unpaired, two-tailed t-test or one-way ANOVA (Prism).
